# Protective Effects of Baicalein on Lipopolysaccharide-Induced AR42J PACs through Attenuation of Both Inflammation and Pyroptosis via Downregulation of miR-224-5p/PARP1

**DOI:** 10.1155/2024/6618927

**Published:** 2024-10-10

**Authors:** Ming-Wei Liu, Chun-Hai Zhang, Shou-Hong Ma, De-Qiong Zhang, Li-Qiong Jiang, Yang Tan

**Affiliations:** ^1^Department of Emergency, Dali Bai Autonomous Prefecture People's Hospital, Dali 671000, China; ^2^Department of Emergency, The First Affiliated Hospital of Kunming Medical University, Kunming 650032, China; ^3^Department of Medical Affairs, The Sixth Affiliated Hospital of Kunming Medical University, Yuxi 653100, China; ^4^Department of Medical Imaging, The First Affiliated Hospital of Kunming Medical University, Kunming 650032, China; ^5^Physical Examination Center, Yunnan Fuwai Cardiovascular Hospital, Kunming 650032, China

## Abstract

**Background:**

Baicalein has been used to treat inflammation-related diseases; nevertheless, its specific mechanism of action is unclear. Therefore, we examined the protective effects of baicalein on lipopolysaccharide-induced damage to AR42J pancreatic acinar cells (PACs) and determined its mechanism of action for protection.

**Methods:**

An *in vitro* cell model of acute pancreatitis (AP) was established using lipopolysaccharide (LPS) (1 mg/L)-induced PACs (AR42J), and the relative survival rate was determined using the 3-(4,5)-dimethylthiahiazo(-z-y1)-3,5-di-phenytetrazoliumromide (MTT) technique. Flow cytometry was applied to evaluate the apoptotic rates of AR42J PACs. The RNA and protein expression of miR-224-5p, poly ADP-ribose polymerase-1 (PARP1), nuclear transcription factor-*κ*B65 (NF-*κ*B65), phospho-kappa B alpha(p-I*κ*B-*α*), interleukin(IL)-18R, NOD-like receptor thermal protein domain-associated protein 3 (NLRP3), gasdermin D (GSDMD), apoptosis-associated speck-like protein containing a CARD (ASC), and caspase-1 was detected based on the WB and RT-PCR assays. IL-1*β*, IL-6, IL-18, and TNF-*α* expression levels in AR42J cells were measured via ELISA method. The cell morphology was examined using the AO/EB method.

**Results:**

The experiment confirmed a significant increase in the activity of AR42J cells treated with various doses of baicalein. Moreover, IL-1*β*, IL-6, TNF-*α*, and IL-18 expression levels in AR42J cells were dramatically reduced (*P*  < 0.05), while miR-224-5p level was obviously enhanced. The protein and gene expression of PARP1, NF-*κ*B65, p-I*κ*B-*α*, IL-18R, GSDMD, ASC, NLRP3, and caspase-1 was obviously decreased (*P* < 0.05). Apoptosis in AR42J cells was significantly reduced with significant improvement in cell morphology.

**Conclusion:**

Baicalein may significantly alleviate LPS-induced AR42J PAC damage by inhibiting the inflammatory response and pyroptosis. Its mode of action might be linked to higher miR-224-5p expression, which inhibits the PARP1/NF-*κ*B and NLPR3/ASC/caspase-1/GSDMD pathways.

## 1. Introduction

Acute pancreatitis (AP) has feature of trypsin activation in pancreatic acinar cells (PACs), leading to self-digestion of the pancreas, PAC injury, systemic inflammatory response syndrome (SIRS), and chronic multiple organ dysfunction [1]. During an AP attack, abnormally activated inflammatory cells overproduce cytokines such as TNF-*α* and interleukins [2, 3]. These inflammatory factors can trigger mediators via pathways like NF-*κ*B and MAPK and caused systemic inflammatory reactions and multiple organ dysfunction syndromes, ultimately increasing mortality in severe pancreatitis patients.

Although AP lacks specific therapy, treatment typically involves inhibiting pancreatic juice and enzyme release and fluid replenishment, correcting electrolyte disorders, providing nutritional support, and balancing acid–base levels. Treatment for severe acute pancreatitis (SAP) is expensive, and outcomes are suboptimal, while mild pancreatitis can resolve spontaneously [1]. However, 20%–30% of mild pancreatitis cases progress to SAP, with a mortality rate of 36%–50% [4]. The diagnosis and therapy of AP were extensively researched both at home and abroad; however, the pathophysiology of AP is not completely understood. Owing to improved standards of living, the incidence of pancreatitis in China is increasing, and the proportion of patients with SAP is also increasing. As a result, clinical research has focused on developing effective therapeutic medications that can enhance SAP survival rates while also lowering treatment costs.

Baicalein (5,6,7-trihydroxy-2-phenyl-4H-benzopyran-4-one) is a crucial active flavonoid. Some scholars found that baicalein has a certain effect on lipopolysaccharide (LPS)-related multitissue injury in mice, including acute lung injury, acute liver failure, myocardial dysfunction, and inflammatory disorders related to endotoxic shock [5, 6, 7]. Baicalein inhibits LPS-induced TNF-*α* formation, NO release, and phosphorylation of I*κ*B-*α* and suppresses the LPS-induced inflammatory response [8]. Baicalein can prevent acinar-to-duct methanogenesis in PACs AR42J and repress the autophagy of PACs induced by sodium taurine cholate [9]. Although baicalein has been shown to lower the serum IL-6 and TNF-*α* concentrations in rat with SAPs, minimize ascites formation, prevent pancreatic damage, and increase the survival rate [10], the mechanism through which baicalein improves AP remains unclear.

PARP-1 is a representative member of the poly (ADP ribose) polymerase family, which contains three domains: an automodification domain, DNA-binding domain, and catalytic domain. The relevant DNA-binding domain contains Zn(I) and Zn(II), which may recognize damaged DNA, whereas Zn(III) is involved in protein binding and activation. The middle automodification domain has a carboxyterminus (for DNA repair and cell signaling) and caspase-3 enzyme cleavage activity. The C-terminal catalytic domain, on the other hand, consists of an *α* helix domain, a tryptophan-glycine-arginine domain (WGR), and an ADP ribotransferase domain. Additionally, the catalytic domain comprises the PARP sequence, which has a 50-amino acid block with complete homology among vertebrates. According to a recent study, PARP-1 serves as a coactivator in NF-*κ*B-mediated transcription by regulating NF-*κ*B and its downstream cytokines TNF-*α* and IL-6, thereby playing a role in inflammation [11].

MicroRNAs (miRNAs) are endogenous, highly conserved noncoding RNAs. Generally, they are about 22 nucleotides long. miRNAs are associated with the degradation and translational suppression of target mRNAs via complementary pairing with the 3′-UTR of target genes. Therefore, this has a negative effect on the posttranscriptional regulation of gene expression [12]. Despite accounting for only 1%–2% of eukaryotic genomes, one miRNA may control many target genes, and the same gene can be controlled by numerous miRNAs, resulting in a complex and sophisticated gene regulation system [12]. As a result, miRNAs have emerged as important regulatory molecules in different pathological processes, like cell growth, differentiation, inflammation, and apoptosis. As revealed in a previous study, miR-224-5p can regulate the apoptosis of breast cancer cells by modulating homeobox A9 and suppressing the development of gastric cancer by regulating the transcriptional activator 6 (ATF6) branches of the unfolded protein response [13]. A recent research found that miR-224-5p overexpression has the potential to inhibit the TLR4/MyD88/NF-*κ*B pathway, thus reducing the ovalbumin-induced allergic rhinitis-associated inflammatory response and thereby limiting allergic rhinitis [14]. Therefore, miR-224-5p may be associated with the onset and progression of AP.

Both PARP-1 and miR-224-5p is a key factor in the occurrence and progression of inflammation and pyroptosis; therefore, we attempted to predict PARP-1 as a potential regulatory target of miR-224-5p based on TargetScan software. In the present research, we postulated that baicalein modulates the LPS-caused inflammatory response in AR42J PACs and reduces LPS-induced damage by altering the miR-224-5p/PARP-1 pathway. This study provides a promising target and a theoretical foundation for the management of AP with baicalein.

## 2. Materials and Methods

### 2.1. Culture and Grouping of AR42J PACs

The PACs AR42J, bought from ATCC, were cultured in RPMI-1640 medium containing 10% FBS. Then, upon attaining a 60% confluence, they were incubated under synchronized conditions for 24 hr before being grouped. Then, it was incubated with the following parameters: 5% CO_2_ at 37°C, subsequently digested with 0.25% trypsin, and cultured for 7 days. The cells were seeded in 25 cm^2^ culture flasks with density of 1 × 10^5^ cells/mL. Upon reaching 60% confluence, they were cultured under synchronized conditions for 24 hr before being grouped. To thoroughly confirm the impact of baicalein on LPS-induced injury in AR42J PACs, cells were initially treated with varied doses of baicalein (25–75 *μ*M) for 2 hr, followed by the addition of 10 mg/L LPS for 24 hr. The expression of miR-224-5p, PARP1, NF-*κ*B, and NLRP3 mRNA and protein was measured via RT-PCR and WB method. The duration of the study ranged from January 15, 2021, to April 14, 2022, and repeated for three times.

### 2.2. Analysis of the Viability of AR42J PACs as Measured by CCK-8

Cell viability of AR42J PACs in every groups was assessed using the CCK-8 kit (Anhui Baioujing Medical Technology Co., Ltd., Anhui, China). AR42J PACs were inoculated onto 96-well plates. Once the cells had reached 70% confluence and changed to serum-free medium, LPS was added at different doses (0.5–2.5 mg/L) for 24 hr, and baicalein (25, 50, or 75 *μ*M) was added to the medium supplemented with 2 or 1 mg/L LPS for 24 hr. After that, 10 *μ*L of CCK-8 solution was added to every well. Afterward, the cells were cultured in dark for ~2–4 hr before being tested for absorbance at 450 nm wavelength.

### 2.3. Evaluation of the Proliferation Inhibition Rate of AR42J PACs

Cell proliferation was evaluated as reference method, with some modifications [15]. AR42J PACs were seeded into 96-well plates containing basic medium at 70% confluence with different doses of LPS (0.5–2.5 mg/L) alone for 24 hr or with baicalein (25, 50, and 75 *μ*M) for 2 hr and subsequently with LPS (1 mg/L) for 24 hr. After 24 hr of incubation, the liquid was removed from each well of the 96-well plates, and then 270 *μ*L of DMEM and 30 *μ*L of MTT working solution (5 g/L) were added. After the resulting mixture was cultured at 37°C for at least 4 min, the intracellular medium was removed, and 270 *μ*L of dimethylsulfoxide was added to form a homogenous solution after shaking for at least 10 min. A microplate reader was applied to detect the OD value of solution at 490 nm. The OD value obtained from the normal group was used as the control. Finally, we calibrated the survival rate (%) of cells using the following formula: OD value of each well/mean OD value × 100 [5].

### 2.4. Cell Transfection

Cell transfection experiments were performed as described previously with fewer alterations [16]. AR42J PACs in the rapid growth stage were seed in 6-well plates. After overnight incubation and reaching 60% confluence, the cells were transfected with miR-224-5p mimic, miR-224-5p inhibitor, miR-224-5p mimic control, and miR-224-5p inhibitor control using Lipofectamine 2000 transfection reagent, according to the relevant instructions. The transfection concentration was maintained at 50 nM. After 6 hr of transfection, the cells were transferred to fresh medium and cultivated for at least 24 hr before being subjected to the WB and RT-PCR analyses.

### 2.5. Analysis of Cell Apoptosis and Mortality

The quantification of cells undergoing apoptosis was performed using flow cytometry, as described earlier, with a few modifications [17]. The well-grown cells were digested, resuspended, and seeded into 6-well plates. In addition, 2 mL of the cell suspension was introduced into each well to cultivate the cells for 24 hr. Subsequently, it was cultured with various LPS doses, namely, 0.5, 1.0, 1.5, 2.0, and 2.5 mg/L, for 24 hr, or with baicalein (25, 50, and 75 *μ*M) for 2 hr and LPS (1 mg/L) for 24 hr. After 24 hr, the suspended cells (apoptotic cells) were transferred to centrifuge tubes, whereas the adherent cells were degraded with trypsin for at least 5 min and then transferred to fresh centrifuge tubes. The cells were rinsed thoroughly via PBS and centrifuged at 5,000 rpm for 6 min to pellet the cells, after which the supernatant was removed. The cell suspension was added to 5 *μ*L of Annexin V and PI for staining without light. The rate of apoptosis and mortality in each group was regarded as the ratio of cells stained with annexin V (Beckman Coulter, Inc., California, USA) according to the corresponding instructions.

### 2.6. Analysis of Cell Morphology and Apoptosis by the Hoechst Method

Cell morphology and apoptosis were analyzed using the Hoechst method as described previously [18]. For this, 3 mL of log-phase-grown AR42J PACs at a cell density of 5 × 10^7^/L were added to the PAC6-well plates and grown overnight with the following set parameters: 37°C, 5% CO_2_, and 60% relative humidity (RH). The cells were grown in various doses of LPS (0.5–2.5 mg/L) for 24 hr or baicalein (25, 50, and 75 *μ*M) for 2 hr and then with LPS (1 mg/L) for 1 day. The adherent cells in each well were rinsed twice via PBS solution. After rinsing with 1 mL PBS, they were then treated with 4% paraformaldehyde and stained via the Hoechst 33258 apoptosis kit (Shanghai Ruji Biotechnology Development Company., Shanghai, China). The cells were visualized via a fluorescence microscope, and images were acquired.

### 2.7. Evaluation of Malondialdehyde (MDA) and NO Contents and SOD Activity

The MDA and NO contents and SOD activity were evaluated as described previously, with a few modifications [19]. AR42J PACs in rapid growth stage were used, thoroughly washed, digested, resuspended, and then seeded in 6-well plates. They were grown overnight in an incubator with the following parameters: 37°C, 5% CO_2_, and 60% RH with various LPS doses (0.5–2.5 mg/L) for 24 hr or baicalein (25–75 *μ*M) for 2 hr and then by LPS (1 mg/L) for 24 hr. After treated, the cells were extracted and subsequently lysed, and the MDA, NO content, and SOD activity were measured according to the manufacturer's instructions (Shanghai Zhuocai Company, Shanghai, China).

### 2.8. Intracellular Reactive Oxygen Species (ROS) Detection

The dihydroethidium fluorescent probe was utilized to detect intracellular ROS, as described previously, with a few modifications [20]. For PACs, log phase-grown AR42J PACs at a density of 5 × 10^7^/L were added to 6-well plates and grown overnight in incubator under condition of 37°C, 5% CO_2_, and RH of 60%. AR42J PACs were cultured with various dose of LPS (0.5, 1.0, 1.5, 2.0, or 2.5 mg/L) for 24 hr or with baicalein (25, 50, or 75 *μ*M) for 2 hr and subsequently with LPS (1 mg/L) for 24 hr. For fluorescence microscopy, the collected cells were resuspended in basic medium supplemented with CM-H_2_DCFDA (10 *μ*mol/L) (Wuhan Boote Biotechnology Co., Wuhan, China) and incubated for 20 min. The incubated samples were inverted at intervals of 5 min to ensure that the CM-H_2_DCFDA fluorescent probe was fully disconnected from the cells. To remove H_2_DCFD, which does not penetrate the cells, it was rinsed with basic culture media once and again with PBS twice. Subsequently, the cells were suspended in 0.5–1 mL of PBS, and the OD value was measured using a fluorescence spectrometer. A 488 nm wavelength was used as the excitation wavelength, while a wavelength of 525 nm served as the emission wavelength. The experiment was repeated three times for every treatment group.

### 2.9. Prediction of the Target Gene

Target gene prediction was performed following a previously described method [21]. Target gene prediction software such as miR-Base, TargetScan, and PicTar has been used to predict that the miR-224 target 5p gene is PARP-1. Conventional methods were utilized to generate wild-type (WT)-NLRP3 and mutant (MUT)-PARP-1 luciferase gene vectors. The above plasmids were cotransfected into AR42J cells with miR-224-5p mimics, miR-224-5p inhibitor, mimic control, and inhibitor control, which were termed the WT + miR-224-5p mimic group, MUT + miR-224-5p group, WT + negative control group, and MUT + negative control group, respectively. Next, the cells were cultivated with standard incubator for 48 hr, and a dual-luciferase reporter gene kit was applied to determine the dual-luciferase activity. The level of NLRP3 in AR42J cells overexpressing miR-224-5p was also tested using the WB and RT-PCR method.

### 2.10. RT Quantitative PCR (RT-PCR)

AR42J cells were harvested, and RNA was extracted using a corresponding extraction kit [22]. The collected and purified RNA was reverse-transcribed into cDNA using the PrimeScript™ RT Reagent Kit (Beijing Zhijie Fangyuan Company, Beijing, China) based on relevant instructions. RT-PCR was applied to determine the expression of PARP-1, IL-18 mRNA, NLRP3, and miR-136–5p. A TaqMan MicroRNA Assay Kit (Biosystems, CA, USA) was applied to detect miR-224-5p levels, whereas the 2^−*∆∆*Ct^ approach was applied to determine the level of miR-224-5p. The sequences of primers used are shown in Table 1. The internal references for miR-136–5p, PARP-1, IL-18, NLRP3, ASC, and caspase-1 were U6 and *β*-actin, respectively. Following the reaction, the Ct of each gene was computed and compared with that of U6 to assess its level.

### 2.11. Western Blot

AR42J PACs in the logarithmic growth phase were used for western blot analysis. The cells were washed, digested, and seeded into 6-well plates, and six replicate wells were established for each group of experiments. The cells were cultured overnight at standard condition in an incubator. Cells were grown with different doses of LPS (0.5–2.5 mg/L) for 24 hr or with baicalein (25, 50, or 75 *μ*M) for 2 hr and LPS (1 mg/L) for 1 day. Finally, the cells were collected and lysed, after which the protein concentrations were calculated. Loading buffer was added to the cell lysate and then heated and boiled at 100°C for 10 min. After electrophoresis, 40 *μ*g of protein was added to each well, and the proteins were separated via 12% SDS-PAGE. The voltage was set at 70 V for stacking gel electrophoresis, whereas it was adjusted to 120 V for separating gel electrophoresis. Following separation, the membrane was transferred for 60 min at 275 mA. The sections were then covered with 5% TBST and left for 1 hr at 25°C. Primary antibodies against PARP1, NF-*κ*B65, P-I*κ*B-*α*, IL-18R, ASC, NLRP3, and caspase-1 (1 : 1,000) (all from Cell Signaling, Inc.) were added to the membrane at 4°C overnight. The membrane was then rinsed via TBST the next day. Next, HRP-labeled goat antirabbit IgG was added (1 : 3,000, Cell Signaling Technology, Inc.), and the procedure was performed at 25°C for 1 hr. The film was then rinsed three times with TBST for 10 min. To visualize the protein bands, an electrochemical luminescence chemiluminescent solution was used. The gray values of the protein bands were determined based on Image software and *β*-actin for reference.

### 2.12. The Levels of IL-6, TNF-*α*, IL-1*β*, and IL-18 in Culture Medium Determined by ELISA

The cell density was adjusted to 1 × 10^8^ L^−1^, and the cells were added to 96-well plates with 0.1 mL every well. Then, they were placed under condition of 5% CO_2_ at 37°C for 24 hr before being treated with baicalein according to the above grouping conditions for 24 hr, after which the cell supernatant was obtained. The expressions of IL-6, TNF-*α*, IL-1*β*, and IL-18 in the cell culture medium were tested using an ELISA kit (iReal Biotechnology, Inc.). A 450 nm value was detected, and the content was estimated based on a standard curve.

### 2.13. Immunofluorescence

AR42J PACs were incubated in 24-well plates; when adhered, they were subjected to drug stimulation for 24 hr. The cells were rinsed with PBS and embedded for 30 min at 25°C. Subsequently, 0.5% Triton X-100 was added to permeabilize the cells for 15 min at 25°C and then blocked at 25°C for 90 min. Primary antibodies (1 : 400 and 1 : 100 dilutions for Ki67 and PCNA, respectively) (all from Elabscience Biotechnology, Inc.) were added and incubated for 14 hr at 4°C. After incubated with a fluorescent secondary antibody (1 : 400 dilution), they were stained with DAPI for 1 hr at 25°C without light. Finally, the position and distribution of Ki67 and PCNA proteins in cells were measured via fluorescence microscopy.

### 2.14. Amylase Activity Measurement

The Nanjing Jiancheng Bioengineering Institute iodine-starch colorimetric kit was used to measure amylase activity, and the determination procedure was carried out as instructed. The following formula was used: culture medium supernatant amylase (U/dL) = (OD of the blank tube − OD of measurement tube)/OD of blank tube × 800.

### 2.15. Statistical Processing

SPSS 17.0 software was applied to statistically treat the data, and all the data were described as mean ± SD. The ANOVA was used to analyze the measurement data. Pairwise comparisons were conducted based on the LSD test. Nonnormally distributed data were transformed into normally distributed data before being subjected to ANOVA. Enumeration data were analyzed via the *χ*^2^ test. *P*  < 0.5 was regarded as statistically judgement criteria.

## 3. Results

### 3.1. Effect of the miR-224-5p Mimic on LPS-Induced AR42J PAC Viability, Inflammation, and Apoptosis

**Figure 1:**
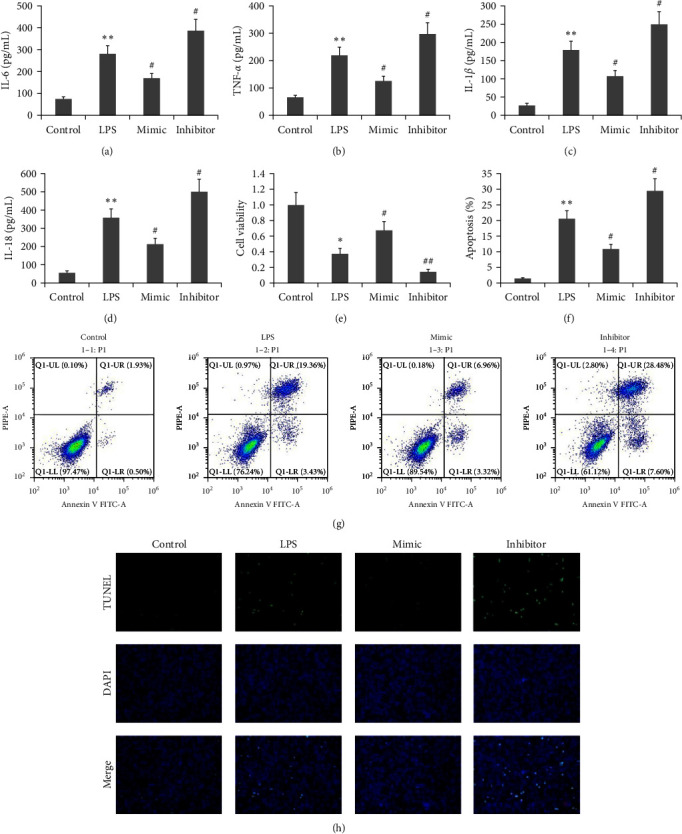
Effect of the miR-224-5p mimic on LPS-induced viability, inflammation, and apoptosis in AR42J PACs. AR42J PACs were either transfected with a miR-224-5p mimic or incubated with an inhibitor for 48 hr in 10 mg/L LPS. (a–d) The levels of IL-6 (a), TNF-*α* (b), IL-1*β* (c), IL-18 (d) were detected via ELISA. (e) The viability of the AR42J PACs was determined based on a CCK-8 assay, and (f–h) AR42J PACs apoptosis status was investigated using flow cytometry (f and g) and Tunel technique (h). The data obtained from three independent experiments are reported as the mean ± SEM.  ^*∗∗*^*P*  < 0.01,  ^*∗*^*P*  < 0.05 versus the control group. ##*P*  < 0.01, #*P*  < 0.01 versus the LPS group.

To clarify if miR-224-5p affects the viability and apoptosis of LPS-induced AR42J PACs, the following two approaches were used: transfecting AR42J PACs with miR-224-5p mimics or the miR-224-5p inhibitor for 48 hr, and incubating them with 10 mg/L LPS. Cell viability was deteceted based on CCK-8 assay. A flow cytometer was used to measure apoptosis. ELISA was used to measure the levels of IL-6, TNF-*α*, IL-1*β*, and IL-18.  Figure 1 illustrates the decrease in apoptosis and proinflammatory cytokine levels of AR42J PACs induced by LPS and the increase in viability levels of AR42J PACs stimulated by LPS when treated with miR-224-5p mimics. In contrast, the miR-224-5p inhibitor increased LPS-induced AR42J PAC apoptosis and inflammation, meanwhile reduced LPS-induced AR42J PAC viability.

### 3.2. Effect of the miR-224-5p Mimic on the PARP1/NF-*κ*B and NLRP3/GSDMD/Caspase-1 Pathways in LPS-Induced AR42J PACs

**Figure 2:**
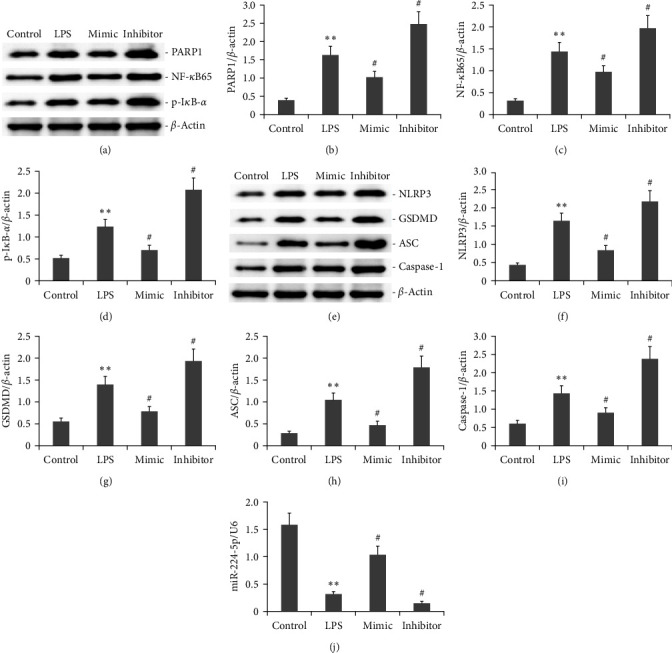
Effect of miR-224-5p on the PARP1/NF-*k*B and NLRP3/GSDMD/Caspase-1 signaling pathway in LPS-induced AR42JPACs. AR42J PACs were either transfected with a miR-224-5p mimic or incubated with a miR-224-5p inhibitor for 48 hr in 10 mg/L LPS. Western blotting was used to measure the protein expression of PACs NLRP3, GSDMD, ASC, and Caspase-1. The protein expression levels of PACs NLRP3, GSDMD, ASC, and Caspase-1 were measured by Western blotting. miR-224-5p expression was measured by RT-PCR. The data obtained from three independent experiments are reported as the mean ± SEM.  ^*∗∗*^*P*  < 0.01 versus the control group. #*P*  < 0.01 versus the LPS group. (a) Typical bands of PARP1, NF-*κ*B65, p-I*κ*B-*α* protein expression. (b–d) Statistical analysis of PARP1, NF-*κ*B65, p-I*κ*B-*α* protein expression. (e) Typical bands of NLRP3, GSDMD, ASC, and Caspase-1 protein expression. (f–i) Statistical analysis of NLRP3, GSDMD, ASC, and Caspase-1 protein expression. (j) RT-PCR analysis of miR-224-5p expression.

To assess the impact of miR-224-5p on the PARP1/NF-*κ*B and NLRP3/GSDMD/caspase-1 signaling pathways in LPS-induced AR42JPACs, these cells were either transfected with a miR-224-5p mimic or miR-224-5p inhibitor for 48 hr and incubating them with 10 mg/L LPS. The WB method was applied to detect PARP1, NF-*κ*B65, p-I*κ*B-*α*, NLRP3, GSDMD, ASC, and caspase-1 protein level in PACs.  Figure 2 exhibits that miR-224-5p mimics decreased the protein levels of PARP1, NF-*κ*B65, p-I*κ*B-*α*, NLRP3, GSDMD, ASC, and caspase-1 in LPS-induced AR42J PACs.

### 3.3. PARP1 Is a Regulatory Target of miR-224-5p

**Figure 3:**
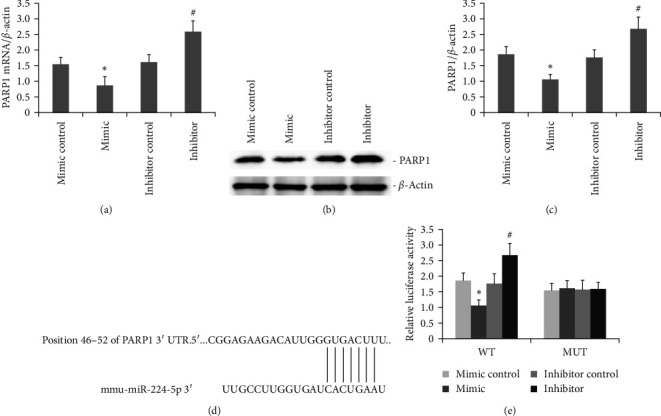
PARP1 is a downstream target of miR-224-5p. (a) Reduced levels of PARP1 mRNA in LPS-induced AR42J PACs following miR-224-5p overexpression (*P* < 0.05). PARP1 mRNA levels were increased following miR-224-5p suppression (*P* < 0.05). (b and c) PARP1 protein levels were lower in LPS-induced AR42J PACs miR-224-5p with overexpression than in those with the empty vector control (*P* < 0.05). PARP1 protein levels are considerably elevated in miR-224-5p downregulated cells. (d) miR-224-5p was bound to the 3′-UTR of PARP1. The binding of mutant PARP1 was disrupted. (e) DLR assays showed that the miR-224-5p mimic bound only to the 3′-UTR of wild-type PARP1 but not to the PARP1 mutants (*P* < 0.05). The data obtained from the three different experiments are reported as the mean ± SEM.  ^*∗*^*P*  < 0.05 versus the mimic control. #*P*  < 0.05 versus the inhibitor control.

The mutual regulatory association in PARP1 and miR-224-5p was analyzed. We utilized TargetScan to identify possible targets of miR-224-5p to clarify how it promotes LPS-induced inflammation and pyroptosis capacity in AR42J PACs. PARP1 is key regulator of LPS-induced inflammation and pyroptosis in AR42J PACs. PARP1 mRNA and protein levels were substantially lower following miR-224-5p overexpression (Figures 3(a), 3(b), and (3(c)). To confirm the link between miR-224-5p and PARP1, we generated WT and mutant PARP1 constructs, and according to the DLR result, miR-224-5p bound to WT PARP1 but not to the mutants, as hypothesized (Figure 3(d) and 3(e)).

### 3.4. Impact of Baicalein on LPS-Caused Inflammation in AR42J PACs

**Figure 4:**
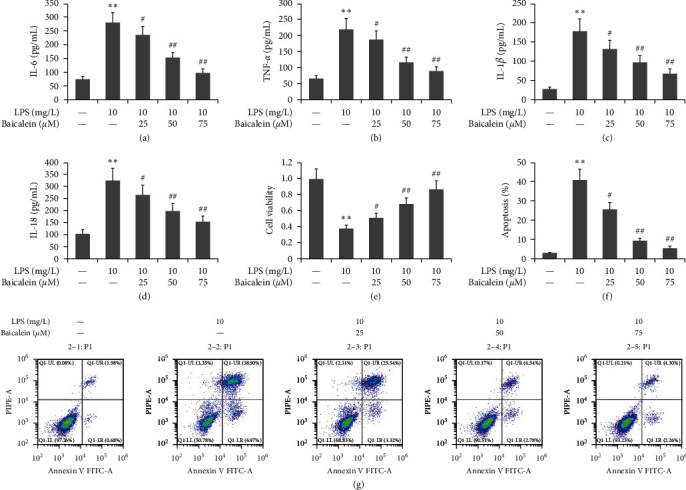
Effect of baicalein on the viability, apoptosis, and inflammation of LPS-treated AR42J PACs. The miR-224-5p mimic was utilized to transfect AR42J PACs in the presence of both 10 mg/L LPS and 50 *μ*M baicalein for 24 hr. ELISAs were used to quantify the levels of IL-6 (a), TNF-*α* (b), IL-1*β* (c), and IL-18 (d) in the cell supernatant. (e) CCK-8 assays were used to measure the viability of the AR42J PACs. (f and g) AR42J PAC apoptosis was established using flow cytometry. The data obtained from the three different experiments are reported as the mean ± SEM.  ^*∗∗*^*P*  < 0.01 versus the control group. ##*P*  < 0.01, #*P*  < 0.01 versus the LPS group.

To study the effect of baicalein on LPS-induced inflammation in AR42J PACs, the cells were treated with LPS (10 mg/L) and different doses of baicalein (25–75 *μ*M) for 24 hr. Meanwhile, ELISA was used to quantify the levels of IL-6, TNF-*α*, and IL-1*β*, as well as IL-18. LPS enhanced the expression of IL-6, TNF-*α*, and IL-1*β*, as well as IL-18 in the cell supernatants of AR42J PACs (Figures 4(a), 4(b), 4(c), and 4(d)). However, baicalein attenuated the expression of them in the cell supernatants of AR42J PACs.

### 3.5. Effect of Baicalein on LPS-Caused Viability and Apoptosis in AR42J PACs

To study the effect of baicalein on these two abilities of LPS-induced AR42J PACs, the cells were treated via LPS (10 mg/L) and different doses of baicalein (25–75 *μ*M) for 24 hr. The viability of the AR42J PACs was determined using a CCK-8 assay. Flow cytometry was used to assess the apoptosis of AR42J PACs. As Figures 4(e) and 4(f), LPS lowered the viability and increased the apoptosis of AR42J PACs in our study; however, it enhanced cell viability while decreasing cell apoptosis.

### 3.6. Effect of Baicalein on the PARP1/NF-*κ*B Pathway in LPS-Caused AR42J PACs by Upregulating miR-224-5p Expression

**Figure 5:**
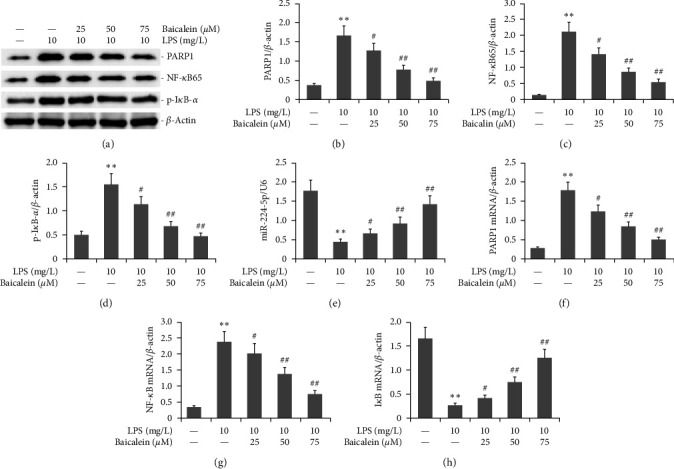
Effect of baicalein on the PARP1/NF-*κ*B signaling pathway in LPS-induced AR42J PACs via the upregulation of miR-224-5p expression. The miR-224-5p mimic was utilized to transfect AR42J PACs in the presence of both 10 mg/L LPS and 50 *μ*M baicalein for 24 hr. (a–d) Western blotting was used to determine the protein expression of PARP1, NF-*κ*B65, and p-I*κ*B-*α*. On the other hand, (e–h) RT‒PCR was used to determine the expression of miR-224-5p, PARP1, NF-*κ*B, and I*κ*B mRNA. The data from three independent experiments are reported as the mean ± SEM.  ^*∗∗*^*P*  < 0.01 versus the control group. ##*P*  < 0.01, #*P*  < 0.01 versus the LPS group.

AR42J PACs were incubated with 10 mg/L LPS and different doses of baicalein (25–75 *μ*M) for 24 hr. PARP1, NF-*κ*B65, and p-I*κ*B-*α* protein level was assessed by western blotting. miR-224-5p, PARP1, NF-*κ*B, and I*κ*B mRNA levels were analyzed via RT-PCR. As demonstrated in Figure 5, LPS increased the protein levels of PARP1, NF-*κ*B65, and p-I*κ*B-*α* in AR42J PACs and downregulated miR-224-5p expression. Conversely, baicalein decreased the levels of PARP1, NF-*κ*B65, and p-I*κ*B-*α* and upregulated miR-224-5p level in AR42J PACs.

### 3.7. Effect of Baicalein on the NLRP3/GSDMD/Caspase-1 Signaling Pathway in LPS-Induced AR42J PACs

**Figure 6:**
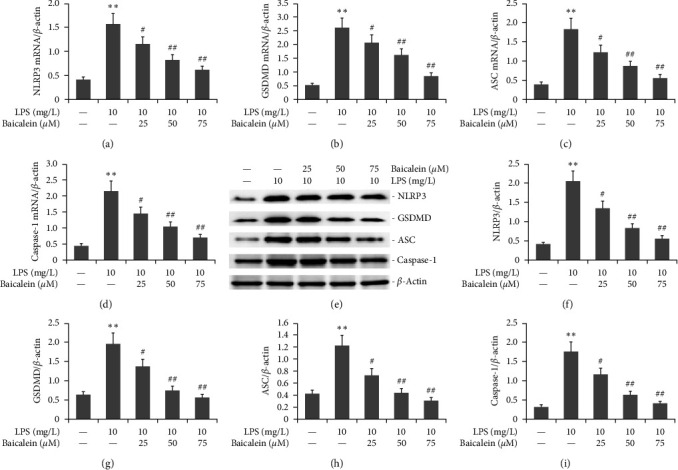
Effect of baicalein on the NLRP3/GSDMD/Caspase-1 signaling pathway in LPS-induced AR42J PACs miR-224-5pvia upregulation of expression. The miR-224-5p mimic was utilized to transfect AR42J PACs in the presence of both 10 mg/L LPS and 50 *μ*M baicalein for 24 hr. PACs. GSDMD, ASC, and Caspase-1 protein expression was analyzed by Western blotting. RT‒PCR was utilized to measure miR-224-5p, GSDMD, ASC, and caspase-3 mRNA expression. The data from three independent experiments are reported as the mean ± SEM.  ^*∗∗*^*P*  < 0.01 versus the control group. ##*P*  < 0.01, #*P*  < 0.01 versus the LPS group. (a–d) Statistical analysis of PACs. GSDMD, ASC, and Caspase-1 mRNA expression. (e) Typical bands of NLRP3, GSDMD, ASC, and Caspase-1 protein expression. (f–i) Statistical analysis of NLRP3, GSDMD, ASC, and Caspase-1 protein expression.

AR42J PACs were treated via LPS (10 mg/L) and different doses of baicalein for 24 hr. NLRP3, GSDMD, ASC, caspase-1 protein, and mRNA levels were assessed by the WB and RT-PCR. As shown in Figure 6, LPS increased the expression of NLRP3, GSDMD, ASC, caspase-1 mRNA, and protein in AR42J PACs. Conversely, baicalein decreased that of NLRP3, GSDMD, ASC, and caspase-1 in AR42J PACs.

### 3.8. Effect of Baicalein on Ki67 and PCNA Expression in LPS-Induced AR42J PACs

**Figure 7:**
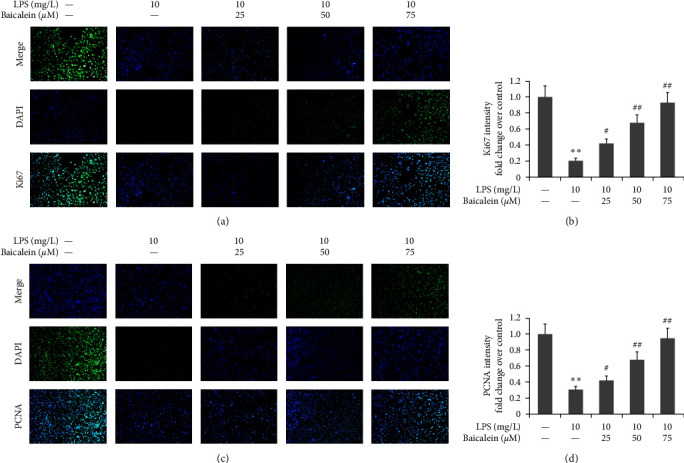
Effect of baicalein on Ki67 and PCNA expression in LPS-induced AR42J PACs.The miR-224-5p mimic was utilized to transfect AR42J PACs in the presence of both 10 mg/L LPS and 50 *μ*M baicalein for 24 hr. (a and b) Ki67- and PCNA-positive expression in LPS-induced AR42J PACs was measured by an immunofluorescence assay. (c and d) Statistical analysis of Ki67- and PCNA-positive expression in LPS-induced AR42J PACs. The data from three independent experiments are reported as the mean ± SEM.  ^*∗∗*^*P*  < 0.01 versus the control group. ##*P*  < 0.01, #*P*  < 0.01 versus the LPS group.

AR42J PACs were treated via LPS (10 mg/L) and different doses of baicalein (25–75 *μ*M) for 24 hr. Ki67- and PCNA-positive expression in LPS-induced AR42J PACs was measured by immunofluorescence. LPS reduced Ki67 and PCNA expression in AR42J cells, as shown in Figure 7. Conversely, baicalein increased both Ki67- and PCNA-positive expression in AR42J PACs.

### 3.9. Effect of Baicalein Combined with miR-224-5p Inhibitor on the PARP1/NF-*κ*B and NLRP3/GSDMD/Caspase-1 Pathways in LPS-Induced AR42J PACs

**Figure 8:**
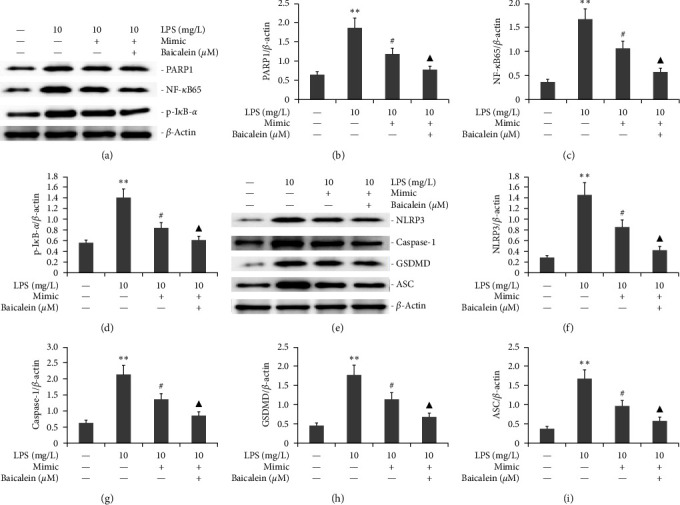
Effect of baicalein combined with miR-224-5p an inhibitor on the PARP1/NF-*k*B and NLRP3/GSDMD/Caspase-1 signaling pathways in LPS-induced AR42J PACs. The miR-224-5p mimic was utilized to transfect AR42J PACs in the presence of both 10 mg/L LPS and 50 *μ*M baicalein for 24 hr. PARP1, NF-*κ*B65, p-I*κ*B-*α*, NLRP3, GSDMD, ASC, and Caspase-1 protein expression was analyzed by Western blotting. The data from three independent experiments are reported as the mean ± SEM.  ^*∗∗*^*P*  < 0.01, ^*∗*^*P*  < 0.05 versus the control group. #*P*  < 0.01 versus the LPS group. ^▲▲^*P*  < 0.01, ^▲^*P*  < 0.01 versus the LPS group. (a) Typical bands of PARP1, NF-*κ*B65, p-I*κ*B-*α* protein expression. (b–d) Statistical analysis of PARP1, NF-*κ*B65, p-I*κ*B-*α* protein expression. (e) Typical bands of NLRP3, GSDMD, ASC, and Caspase-1 protein expression. (f–i) Statistical analysis of NLRP3, GSDMD, ASC, and Caspase-1 protein expression.

The miR-224-5p mimic was used to transfect AR42J PACs with the existence of both 10 mg/L LPS and 50 *μ*M baicalein for 24 hr. PARP1, NF-*κ*B65, p-I*κ*B-*α*, NLRP3, GSDMD, ASC, and caspase-1 protein expression was analyzed by western blotting method. As demonstrated in Figure 8, the miR-224-5p inhibitor increased the protein levels of PARP1, NF-*κ*B65, p-I*κ*B-*α*, NLRP3, GSDMD, ASC, and caspase-1 in AR42J PACs. Conversely, baicalein combined with a miR-224-5p inhibitor decreased the protein level of PARP1, NF-*κ*B65, p-I*κ*B-*α*, NLRP3, GSDMD, ASC, and caspase-1.

### 3.10. Effects of Baicalein Combined with miR-224-5p Mimic on LPS-Caused Inflammation, Viability, and Apoptosis in AR42J PACs

**Figure 9:**
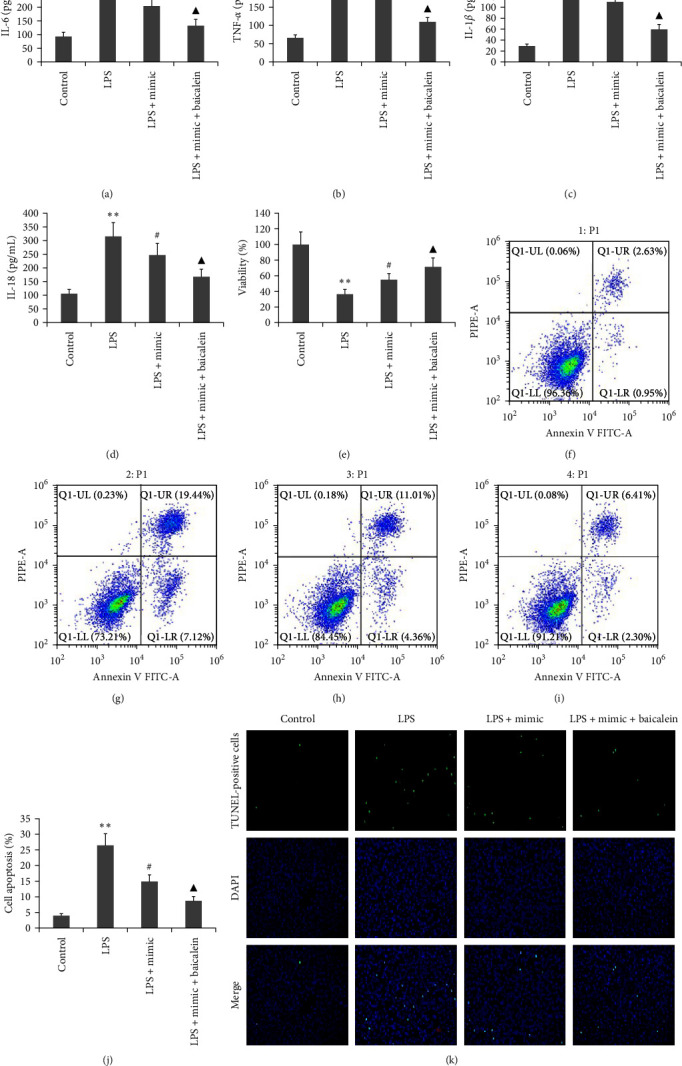
Effect of baicaleinin in combination with the miR-224-5p mimic on LPS-induced inflammation, viability, and apoptosis in AR42J PACs. The miR-224-5p mimic was utilized to transfect AR42J PACs in the presence of both 10 mg/L LPS and 50 *μ*M baicalein for 24 hr. (a–d) The levels of IL-6, TNF-*α*, IL-1*β*, and IL-18 in the cell supernatant were measured by ELISA. (e) The viability of AR42J PACs was determined with a CCK-8 assay. (f–k) The apoptotic rates was ascertained with the aid of flow cytometry and TUNEL technique. The data from three independent trials are reported as the mean ± SEM.  ^*∗∗*^*P*  < 0.01 versus the control group. ##*P*  < 0.01, #*P*  < 0.01 versus the LPS group. ^▲▲^*P*  < 0.01, ^▲^*P*  < 0.01 versus the LPS group.

The miR-224-5p mimic was used to transfect AR42J PACs with the existence of both 10 mg/L LPS and 50 *μ*M baicalein for 24 hr. The levels of IL-6, TNF-*α*, IL-1*β*, and IL-18 were detected via ELISA. The viability of the AR42J PACs was determined based on a CCK-8 assay, and their apoptosis status was investigated using flow cytometry. As shown in Figure 9, the miR-224-5p mimic decreased the levels of IL-6, TNF-*α*, IL-1*β*, and IL-18 in supernatant, and the apoptosis of the AR42J PACs increased the viability of the AR42J PACs. Baicalein combined with the miR-224-5p mimic further decreased the levels of them in the cell supernatant and decreased the apoptosis of AR42J PACs.

### 3.11. Effect of Baicalein Combined with miR-224-5p Mimic on the Cellular Morphology and Proliferation of LPS-Induced AR42JPACs

**Figure 10:**
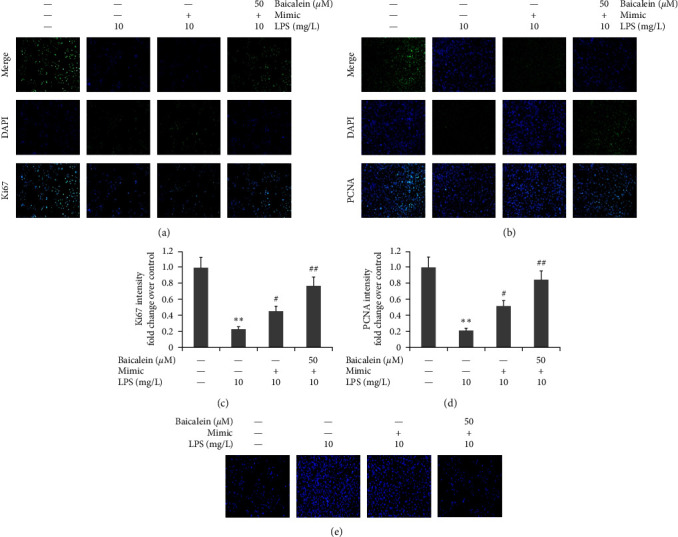
Effect of baicalein mixed with miR-224-5p a mimic on the cellular morphology of LPS-induced AR42J PACs. The miR-224-5p mimic was utilized to transfect AR42J PACs in the presence of both 10 mg/L LPS and 50 *μ*M baicalein for 24 hr. (a–d) Ki67- and PCNA-positive expression in LPS-induced AR42J PACs was measured using immunofluorescence. (e) Cellular characteristics were ascertained by the Hoechst method. The data from three independent experiments are reported as the mean ± SEM.  ^*∗∗*^*P*  < 0.01 versus the control group. ##*P*  < 0.01, #*P*  < 0.01 versus the LPS group.

The miR-224-5p mimic was used to transfect AR42J PACs with the existence of both 10 mg/L LPS and 50 *μ*M baicalein for 1 day. Cellular morphology was detected via the Hoechst method. Ki67- and PCNA-positive expression in LPS-induced AR42J PACs was measured using immunofluorescence. As shown in Figure 10, Hoechst 33258 staining revealed that the control group cells were uniform in shape and size, with clear borders, and no obvious morphological changes in the nucleus, showing weak normal blue fluorescence. After LPS treatment, the cells were irregular in shape and swollen, and the organelles were distorted or expanded, with nuclear lysis and chromatin condensation. AR42J cell proliferation was increased dramatically, and the morphology of LPS-induced AR42J cells damage was improved dramatically following baicalein treatment.

## 4. Discussion

AR42J cells are pancreatic exocrine cells of rats, and their receptor expression and signal transduction are the same as those of normal PACs. Cell culture is not completely subjected to other interference, and the experimental system is simple and provides favorable culture conditions, high transfection efficiency, and sensitivity to stimulation [23]. LPS, an integral constituent of gram-negative bacterial cell walls, plays a role in the pathogenesis of AP [24]. LPS can transform moderate to mild pancreatitis into severe pancreatitis and subsequently cause systemic inflammatory syndrome [24]. LPS can induce and stimulate AR42J cells to produce numerous inflammatory factors with high repeatability, which is beneficial in an *in vitro* cell model for studying pancreatitis [24]. Hence, in the present research, LPS was applied to stimulate AR42J cells to construct a pancreatitis cell model.

The pathophysiology of AP is complicated, and it is believed that the inflammatory reaction in PACs caused by the abnormal activation of pancreatin is a key cause of this disease. In the early stages of pancreatitis, PACs produce inflammatory factors and chemokines, which can damage them and trigger a systemic inflammatory response, leading to multiple organ dysfunction. LPS has been shown to boost the production of IL-6, TNF-*α*, IL-1*β*, ICAM-1, and IL-18 by PACs, suggesting that LPS can increase the inflammatory response of PACs. Some studies have shown that low-dose LPS can trigger acinar cell apoptosis, but high-dose LPS can cause cell edema, damage the cytoplasmic membrane and lysosome, and increase amylase secretion [25, 26], which is consistent with our findings. NF-*κ*B is extensively involved in inflammatory and immune responses. NF-*κ*B was a key factor in the pathophysiology of pancreatitis. In our study, stimulation of acinar AR42J cells with LPS activated the NF-*κ*B pathway, enhanced acinar cell AR42J inflammation, inhibited acinar cell proliferation, and triggered cell death. However, baicalein treatment suppressed the expression of NF-*κ*B pathway, which partially reversed the effects of LPS stimulation.

As confirmed by previous studies, simultaneous activation of the inflammatory response and PARP-1 occurs in many inflammatory disorders, and methods to suppress PARP-1, such as gene deletion and inhibitor injection, can ameliorate the inflammatory response and disease development [27, 28]. During severe infection, PARP overexpression can modulate the ability of the pleiotropic transcriptional regulator NF-*κ*B to penetrate the nucleus, bind to its corresponding site, and promote the release of its downstream cytokines. This further activates NF-*κ*B to form positive feedback, release a large number of inflammatory factors, form a chain of cytokine network cascades, damage the intestinal mucosal barrier and ectopic flora, cause SIRS and MODS, and even result in death [29, 30]. In this study, LPS upregulated PARP1 and NF-*κ*B expression. Therefore, the downregulation of PARP-1 expression attenuated the NF-*κ*B release and the LPS-induced inflammatory response in PACs. As revealed by some studies, appropriate doses of PARP-1 inhibitors, such as 3-AB, can significantly alleviate pancreatic pathological damage in SAP mice and decrease serum amylase, lipase, IL-1, and IL-6 expression levels, and investigations have shown that inhibiting PARP-1 can improve the course of SAP [31, 32]. Compared with the severity of AP in WT mice, the severity of AP in mice lacking PARP-1 (PARP-1^−/−^) was obviously decreased, as was the severity of SAP-related lung damage [31]. After the administration of PJ34, 3-AB, or other inhibitors, pancreatitis and lung-related damage in SAP mice were markedly alleviated, suggesting that decreasing or decreasing PARP1 expression can minimize LPS-induced AP damage and lung damage. In the present study, baicalein successfully decreased the level of PARP1 and the activity of NF-*κ*B pathway and the LPS-induced inflammation and pyroptosis of acinar cells.

A recent study confirmed that factors related to the classical NLPR3/ASC/caspase-1/GSDMD pathway are involved in pyroptosis, as they are highly expressed in the cells of both humans and animals with sepsis, and the use of blockers or knockouts of related factors in this pathway can attenuate the systemic inflammatory response, thus preventing damage to organ function to a certain extent [33]. Undercondition of the NLRP3 inflammasome is activated, and it can activate caspase-1, which then cleaves gasdermin D (GSDMD) and perforate the cell membrane and formed a pore. Membrane rupture, cell osmolysis, DNA lysis, and the release of cell contents and IL-1*β* and IL-18 caused strong inflammatory response [34, 35, 36]. ASC acts by bridging NLRP proteins, such as NLRP1, which caused the activation of caspase-1 and promote the release of IL-1*β* and IL-18 [37]. In the present research, it was found that LPS may promote the protein expression of NLPR3, caspase-1, and ASC, as well as the production of the inflammatory mediators in pancreatic acinar cells, suggesting that LPS may promote pyroptosis. Wang et al. [38] showed that the TLR4/NF-*κ*B pathway can induce GSDMD-mediated renal tubular epithelial cell pyroptosis in diabetic nephropathy. Another study by Liu et al. [39] revealed that melatonin can attenuate the pyroptotic response of cells by blocking the NF-*κ*B/GSDMD signaling pathway in mouse adipose tissue. In the present research, it was found that LPS increased the activity of the PARP1 and NF-*κ*B pathways, which in turn increased the activity of the NLPR3/ASC/caspase-1/GSDMD pathways, thereby increasing PAC inflammation and pyroptosis. Baicalein treatment repressed the expression of the PARP1 and NF-*κ*B pathways and partially reversed LPS-caused inflammation and pyroptosis in pancreatic acinar cells by decreasing the activity of the NLPR3/ASC/caspase-1/GSDMD pathway.

By targeting PTEN, miR-224-5p regulates pancreatic mucinous cystadenocarcinoma cell proliferation and invasion [40]. Similarly, the downregulation of miR-224-5p expression enhances stem cell migration in human dental pulp, whereas the upregulation represses these processes [41]. miR-224-5p, which controls the autophagy of malignant breast cells via the homeobox protein Hox-A5 [42], is found in exosomes produced from mesenchymal stem cells of the human umbilical cord. miR-224-5p shields dental pulp stem cells from death by acting on Rac1 [43]. According to a recent study, miR-224-5p can negatively modulate inflammatory responses. The overexpression of miR-224-5p suppressed caspase 1, NLRP3, and IL-1*β* expression *in vitro* [44]. miR-224-5p has a protective effect on microglia, as it can attenuate the activation of microglial inflammation by modulating the expression of NLRP3 [44]. According to our study, LPS decreased miR-224-5p levels and increased the activity of the PARP1/NF-*κ*B and NLPR3/ASC/caspase-1 pathways, thereby increasing pancreatic acinar cell inflammation and pyroptosis. Moreover, baicalein treatment increased miR-224-5p expression, causing repression of both the PARP1 and NF-*κ*B pathways and thereby reversing LPS-induced inflammation and pyroptosis in such cells.

In this study, both miR-224-5p and PARP1 were found to modulate inflammation and pyroptosis in such kind cells. In AR42J PACs treated with the miR-224-5p inhibitor, the expression of miR-224-5p decreased, whereas that of PARP1 increased. Similarly, when transfected the miR-224-5p mimic into AR42J cells, we discovered that the level of miR-224-5p was upregulated, and that of PARP1 was downregulated. Based on these observations, it is speculated that miR-224-5p may mediate LPS-induced inflammation and pyroptosis in acinar cells by targeting PARP1. To establish a link in miR-224-5p and PARP1 expression, PARP1 was identified as a regulatory target of miR-224-5p using the TargetScan program. The WT and mutant type (MUT) PARP1 constructs were prepared, and DLR assays were subsequently performed. miR-224-5p was found to bind to WT-PARP1 but not to MUT-PARP1, providing strong evidence confirming that PARP1 is a regulator of the target of miR-224-5p. These findings demonstrate that miR-224-5p can regulate the activity of the NF-*κ*B pathway by impacting the expression of PARP1, consequently modulating the activity of the NLPR3/ASC/caspase-1/GSDMD pyroptosis pathway, thereby regulating LPS-induced inflammation and pyroptosis in acinar cells.

Baicalein has also been previously reported to suppress the stimulation and nuclear translocation of STAT in LPS-stimulated RAW264.7 cells [45]. These results indicated that baicalein is a promising drug for managing LPS-caused inflammatory illness. Recently, Li et al. [46] utilized LPS-caused RAW264.7 cells as an inflammation model and revealed that baicalein may quickly inhibit the release of TNF-*α* in a dose-dependent manner, resulting in a reduction in the levels of IL-6 and iNOS/NO, as well as a decrease in the inflammatory response. Another study [8] illustrated that baicalein could alleviate colitis symptoms by inhibiting the TLR4/MyD88 signaling cascade and activating the NLRP3 in mice.

Pu et al. [47] suggested that baicalein may limit the metaplasia of ductal cells by blocking the inflammatory response of LPS-activated acinar cell line AR42J, preventing the stimulation of NF-*κ*B, and subsequently reducing the number of inflammatory factors. In the present research, we revealed that baicalein may increase the miR-224-5p level, subsequently controlling PARP1 activity and blocking the NF-*κ*B pathway while reducing NLPR3/ASC/caspase-1/GSDMD pyroptosis pathway activity, thereby suppressing LPS-caused inflammation in acinar cells.

Our research revealed that the mechanism is related to the fact that baicalein suppressed LPS-caused inflammation in acinar cells by increasing the activity of the miR-224-5p/PARP1/NF-*κ*B pathway and reducing the activity of the NLPR3/ASC/caspase-1/GSDMD pyroptosis pathway. This paper still has room for improvement. For example, additional animal experiments were not performed. Second, we did not investigate the 50% effective or inhibitory dose or the minimum inhibitory concentration of baicalein in the baicalein-treated acinar cell line AR42J. The protective function of baicalein on endotoxin-caused AR42J in acinar cells may involve inflammation, autophagy, pyroptosis, and ferroptosis. Therefore, further research is required to clarify the mechanisms involved. Furthermore, no further miRNA target genes were identified in this investigation. miR-224-5p may influence endotoxin-induced acinar cells via additional signaling mechanisms that can be investigated in future studies. These results can confirm the same findings in *in vivo* models [48] and provide support for relevant research.

## 5. Conclusion

**Figure 11:**
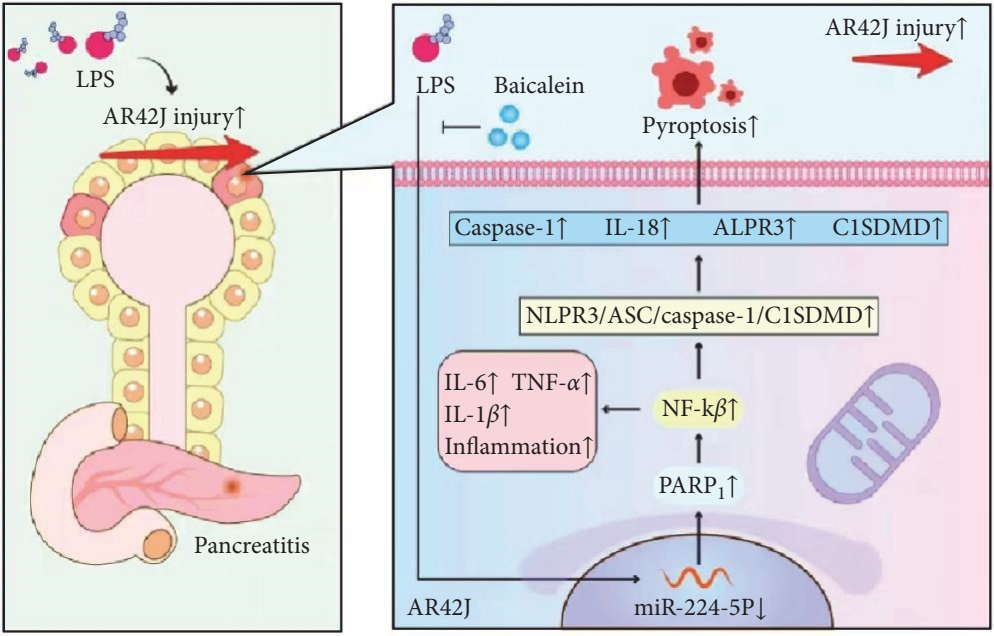
The mechanisms underlying the protective effects of baicalein on LPS-induced AR42J PACs.

Therefore, this research confirmed that miR-224-5p can modulate LPS-caused inflammation and pyroptosis in PACs by targeting the expression of PARP1 (Figure 11). Baicalein can ameliorate LPS-induced pancreatic acinar cell damage by suppressing the LPS-induced inflammatory response and pyroptosis of PACs (Figure 11). Baicalein can increase the level of miR-224-5p and repress the activity of the inflammatory pathway PARP1/NF-*κ*B and pyroptosis pathway NLPR3/ASC/caspase-1/GSDMD (Figure 11).

## Figures and Tables

**Table 1:** RT-PCR primer sequences.

Gene	Primer
miR-224-5p	F-5′-AGCTGGTGTTGTGAATCAGGCCG-3′
	R-5′-TGGTGTCGTGGAGTCG-3′
U6	F-5′-CTCGCTGCCGACTGAGA-3′
	R-5′-AACTGCCGACTGAGGCGT-3′
PARP1mRNA	F-5′-AGTGATTGGCAGCAGGTC-3′
	R-5′-GGTGTCTGGGATGTTTAGG-3′
NLRP3 mRNA	F-5′-CCCCGTGAGTCCCATTA-3′
	R-5′- GACGCCCAGTCCAACAT-3′
ASC mRNA	F-5′-GACGCCTTGGACCTCACCGA-3′
	R-5′- TTGGCTGCCGACTGAGGAGG-3′
GSDMD mRNA	F-5′-CAGGCAGCATCCTTGAGTGTC-3′
	R-5′- CCAAGACGTGCTTCACCAACT-3′
Caspase1 mRNA	F-5′-AGACTTCTGACAGTACCTTCCTTG-3′
	R-5′-GCCTTCTTAATGCCATCATCTTCAA-3′
I*κ*B mRNA	F-5′-AACAGCTGCCTCTCCAAGAT-3′
	R-5′-TGTCCGATGTGAGTCCAAAC-3′
NF-*κ*B mRNA	F-5′-TGCGATCCGCTAAATGCGA-3′
	R-5′-AGGCATTCATGTGGATAGGCTA-3′
*β*-Actin	F-5′-ATGGTGAAGGTCGGTGTG-3′
	R-5′-AACTTGCCGTGGGTAGAG-3′

## Data Availability

The datasets utilized and analyzed in this research can be obtained upon request from the author.

## Abbreviations

MiR-224-5p: MicroRNA-224-5p
PARP-1: Poly ADP-ribose polymerase-1
LPS: Lipopolysaccharide
NF-*κ*B: Nuclear factor kappa
p-I*κ*B-*α*: Phospho-kappa B alpha
NLRP3: Nucleotide-binding oligomerization domain-like receptor 1
SIRS: Systemic inflammatory response syndrome
UTR: 3′-Untranslated region
PACs: Pancreatic acinar cells
ASC: Apoptosis-associated speck-like protein
GSDMD: Gasdermin D
TLR4: Toll-like receptor 4
IL-10: Interleukin-10
TNF-*α*: Tumor necrosis factor-*α*
IL-1*β*: Interleukin-1*β*
IL-18: Interleukin-18
MTT: 3-(4,5)-Dimethylthiahiazo(-z-y1)-3,5-di-phenytetrazoliumromide
IL-6: Interleukin-6
RT-PCR: Reverse transcription polymerase chain reaction
DTR: Dual-transferase reporter
CCK-8: Cell Counting Kit-8
WGR: Tryptophan-glycine-arginine domain
ADP: Ribotransferase domain
CARD: Caspase recruitment domai.
